# Application of Adzuki Bean Starch in Antioxidant Films Containing Cocoa Nibs Extract

**DOI:** 10.3390/polym10111210

**Published:** 2018-10-31

**Authors:** Sujin Kim, Su-Kyoung Baek, Eunjeong Go, Kyung Bin Song

**Affiliations:** Department of Food Science and Technology, Chungnam National University, Daejeon 34134, Korea; pppink32@gmail.com (S.K.); sukyoungb@naver.com (S.-K.B.); ko8546882@naver.com (E.G.)

**Keywords:** adzuki bean, antioxidant activity, biodegradable films, cocoa nibs extract

## Abstract

In this study, starch extracted from adzuki bean (ABS) was used as a biodegradable film source. In addition, to develop a new antioxidant film, various amounts of cocoa nibs extract (CNE, 0.3%, 0.7%, and 1%) were incorporated. With the addition of CNE, the elongation at break of the ABS films increased and the tensile strength decreased. The ABS films with CNE showed increased 2,2′-azino-bis-3-ethylbenzthiazoline-6-sulphonic acid (ABTS) and 1,1-diphenyl-2-picrylhydrazyl (DPPH) scavenging activities with increasing amounts of CNE. In particular, the ABTS and DPPH radical scavenging activities of the ABS films containing 1% CNE were 100% and 94.9%, respectively. Furthermore, decomposition of the films was observed after 28 days of biodegradation. Thus, ABS films containing CNE can be applied as a new active packaging material.

## 1. Introduction

The use of synthetic polymers in the food packaging industry has increased because of their convenience. However, synthetic polymers cause environmental problems, as they are difficult to decompose naturally. Thus, interest in eco-friendly biopolymers as a substitute for synthetic polymers has increased [1]. Starch is widely studied as a base material for biodegradable films because it is one of the most abundant land resources and has good film-forming ability and biodegradability [2]. Among starches, cassava, corn, and potato have been widely used, but few studies have been conducted to develop films based on under-utilized starch sources.

Adzuki bean (*Vigna angularis*) is a traditional legume that has been cultivated throughout East Asia for a long time [3] and is the second most economically important legume in Japan [4]. Adzuki bean contains approximately 60% starch in its composition, and the amylose content of adzuki bean starch (ABS) is estimated at around 20% to 30% [3,5]. Despite its high starch and amylose content, the usage of ABS in the food industry has been limited and ABS has not been reported as a film base material.

Biodegradable films have been applied in various active and smart packaging systems [6]. Among them, active packaging can be developed by adding active materials with antioxidant activity. The incorporation of antioxidants in the films has advantages over direct addition to foods because the antioxidants can be gradually released into packaged food, resulting in extension of shelf life by delaying lipid oxidation in the foods. Antioxidants can be obtained synthetically or naturally. However, as consumer interest in health has increased, natural antioxidants have received more attention than synthetic preservatives. Many studies have focused on natural antioxidants, such as yerba mate, rosemary, and tea extracts [7,8,9].

Recently, there has been growing interest in polyphenols due to their health benefits, including antioxidant, anti-inflammatory, and anti-atherogenic effects [10]. Cocoa nibs, which are derived from *Theobroma cacao* L., have been extensively studied. In particular, cocoa nibs extract (CNE) is a good source of phenolic compounds, principally flavan-3-ol compounds such as catechin and epicatechin [10,11]. Among natural antioxidants, CNE exhibits higher antioxidant activity (253.9 µmol of TE/g) than blackberry extract (55.7 µmol of TE/g) or cranberry extract (18.5 µmol of TE/g [12,13]. However, at present there are no reports on biodegradable films containing CNE as a natural antioxidant. Therefore, the present study aimed to develop and characterize antioxidant starch films from ABS and CNE, which have not been applied in the food packaging industry.

## 2. Materials and Methods

### 2.1. Materials

Adzuki bean (Cheonaeji, Incheon, Korea), sorbitol, gallic acid, and epicatechin (Sigma-Aldrich Co., St. Louis, MO, USA) were used in this study. Cocoa nibs were obtained from Gilim Co., Ltd. (Gwangju, Korea).

### 2.2. Extraction of ABS

ABS was prepared according to the method described by Hoover et al. [5] with minor modifications. Adzuki bean powder and 0.3% NaOH solution (1:5, *w*/*v*) were mixed and stirred (4 °C, 24 h). The solution was then passed through a 200-mesh sieve and centrifuged at 3800× *g* for 10 min. Afterwards, the pellet was mixed in distilled water (DW) and neutralized with 1 N HCl solution. The slurry was kept at 4 °C for 1 h and the pellet was dried in a drying oven (DDO-102, Daeil Engineering Co., Seoul, Korea) at 50 °C for 24 h. Starch yield was calculated based on adzuki bean flour (dry basis). The yield of the starch was 40%, which was higher than that reported in the literature [5].

### 2.3. Extraction of CNE

CNE was extracted from cocoa nibs as described by Mazor Jolić et al. [12] with minor modifications. Cocoa nibs (10 g) and 70% ethanol (100 mL) were mixed and stirred at 25 °C for 5 h. Whatman filter paper No. 2 was then used for filtration, and the filtrate was concentrated with a vacuum evaporator and dried using a freeze-drier (FD5508, Ilshin Lab Co., Yangju, Korea).

### 2.4. Amylose Content of ABS

The amylose content of ABS was determined [14]. ABS powder (100 mg), 95% ethanol (1 mL), and 1 N NaOH solution (9 mL) were mixed and heated in a water bath at 80 °C for 10 min. After cooling down for 15 min, DW was added and the solution (5 mL), 1 N acetic acid (1 mL), and I2-KI solution (2 mL) were mixed and topped with DW to 100 mL. The mixture was kept at 25 °C for 20 min and the absorbance was measured at 620 nm using a spectrophotometer (UV-2450, Shimadzu Corporation, Kyoto, Japan).

### 2.5. CNE Analysis

The CNE composition was analyzed using a high-performance liquid chromatography (HPLC) system (Waters Corporation, Milford, MA, USA) using an Xbridge ^TM^ C18 column (4.6 mm × 50 mm, Waters Corporation, Milford, MA, USA) according to the method described by Schinella et al. (2010). Prior to injection into the column, CNE and standard chemical (epicatechin) were dissolved in methanol and passed through a polyvinylidene fluoride filter (0.45 µm, Whatman^TM^, Clifton, NJ, USA).

### 2.6. Preparation of ABS Films Containing CNE

To prepare the ABS films containing CNE, ABS (2.5%) and sorbitol (0.4 g/g ABS) were first dispersed in DW and stirred at 80 °C for 20 min. Various amounts (0.3%, 0.7%, and 1%) of CNE were then added to the film-forming solution and sonicated for 5 min. The filtered film solution (30 mL) was uniformly spread onto glass plates and dried on a clean bench at 25 °C for 15 h. The control film was prepared using ABS and sorbitol only without the addition of CNE.

### 2.7. Physical Properties

The physical properties of the ABS films were examined using a Testometric machine (250-2.5 CT, Testometric Co., Lancashire, UK). The water vapor permeability (WVP), water solubility (WS), and moisture content (MC) of the ABS films were measured as described by Yang et al. [15]. Five replications were recorded independently.

### 2.8. Color Measurement and Ultraviolet (UV)-Visible Light Transmittance

To analyze the color of the ABS films, a colorimeter (CR-400, Minolta, Tokyo, Japan) was used. According to the CIELAB color scale, the *L* * (lightness), *a* * (redness), and *b* * (yellowness) values were examined. In addition, the opacity of the ABS films was determined using a spectrophotometer (UV-2450, Shimadzu Corporation, Kyoto, Japan) [16]. To analyze the UV-visible light transmittance of the ABS films in the range of 200 to 800 nm, a spectrophotometer (UV-2450, Shimadzu Corporation) was used.

### 2.9. Total Phenolic Content (TPC)

The TPC of the ABS films was estimated according to the method described by Adilah et al. [17], and gallic acid was used as a standard.

### 2.10. Antioxidant Activity

Assays of 2,2′-azino-bis-3-ethylbenzthiazoline-6-sulphonic acid (ABTS) and 1,1-diphenyl-2-picrylhydrazyl (DPPH) radical scavenging were performed to examine the antioxidant activity of ABS films containing CNE [16].

### 2.11. Attenuated Total Reflectance-Fourier Transformation Infrared (ATR-FTIR) Spectroscopy

An FT-IR spectrophotometer (VERTEX 80v, Bruker Optics, Billerica, MA, USA) was used to obtain the ATR-FTIR spectra of ABS films containing CNE in the wavenumber range of 4000 to 400 cm^−1^.

### 2.12. Differential Scanning Calorimetry (DSC) and Thermogravimetric Analysis (TGA)

DSC and TGA were conducted using a differential scanning calorimeter (DSC1; Mettler Toledo, Columbus, OH, USA) at a rate of 10 °C/min. Each sample was scanned from 0 to 200 °C for DSC and heated from 25 to 700 °C for TGA.

### 2.13. Biodegradation Test

The biodegradability of the ABS films was evaluated according to the method described by Assis et al. [18] with minor modifications. Each film (2 cm × 2 cm) was weighed and buried in the vegetable compost inside a plastic tray (15 cm × 25 cm). The trays were kept at 25 °C and 60% relative humidity for 28 days. After 4, 7, 14, 21, and 28 days, the films buried in the soil were weighed.

### 2.14. Statistical Analysis

SAS software (SAS Institute Inc., Cary, NC, USA) was used to evaluate the experimental results. Statistically significant difference was determined at *p* < 0.05. All values are presented as mean ± standard deviation. Each experiment was replicated at least three times.

## 3. Results and Discussion

### 3.1. Starch Yield and Amylose Content

The yield of the ABS was 40%, which was higher than that reported in the literature. According to the report by Hoover et al. [5], the ABS yield was 21.5%. The variation of starch yield could be attributed to a different cultivar and physiological state of the seed [5].

The amylose content in starch is important because it can affect the chemical properties of starch and can determine its application [19]. Particularly, for starch-based films, the amylose–amylopectin ratio affects the physical properties of the films because the starch network and gel properties of starch-based polymers are altered [20]. In general, starch with high amylose content renders good physical properties in the starch films. Nogueira et al. [19] reported that amylose content was a determinant of the physical properties of arrowroot starch films. In the present study, the amylose content of ABS was 22.1%, which is similar to a previously reported value [5].

### 3.2. HPLC Analysis of CNE

**Figure 1:**
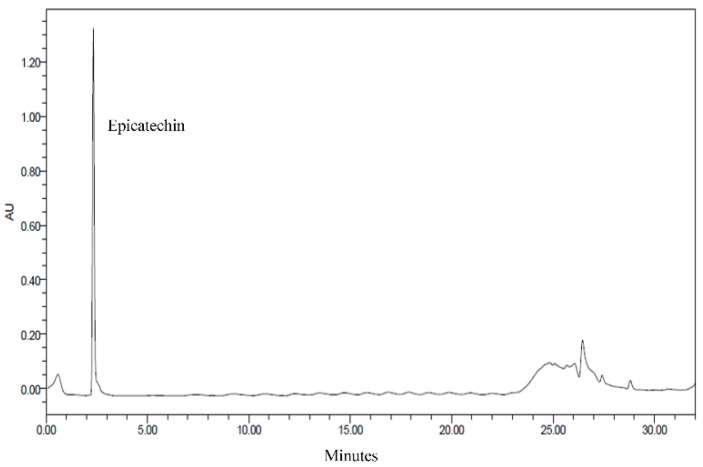
High-performance liquid chromatography (HPLC) chromatogram of cocoa nibs extract.

CNE contains a considerable amount of epicatechin, which has strong antioxidant activity [11,21]. Epicatechin is a major component of the CNE prepared in this study (Figure 1). Thus, the antioxidant capacity of CNE is mainly due to epicatechin. Similarly, Schinella et al. [10] reported the high antioxidant activity of CNE with epicatechin as the major component, assessed using HPLC.

### 3.3. Physical Properties of ABS Films Containing CNE

The thickness, tensile strength (*TS*), elongation at break (*E*), and WVP of ABS films containing CNE are presented in Table 1. The incorporation of CNE decreased the rigidity of the ABS films and increased their flexibility. In particular, the ABS film without CNE exhibited a *TS* of 30.59 MPa, whereas those of the films incorporated with various amounts of CNE (0.3%, 0.7%, and 1%) decreased to 27.74, 25.50, and 18.58 MPa, respectively. In contrast, the *E* values of the ABS films increased from 10.11% to 27.80% when the amount of CNE increased from 0% to 1%. These results suggest that the incorporation of CNE into the ABS films led to structural changes, resulting in a decrease in intermolecular interactions among starch molecules. These findings are consistent with the results of Kuorwel et al. [22], whereby a decrease in the *TS* of thermoplastic starch films was observed by the addition of 3.76% thymol or 3.49% carvacrol. Similarly, Bof et al. [23] reported that the *TS* of starch/chitosan films decreased from 18.4 to 7.9 MPa by the addition of grapefruit seed extract, and it was suggested that the extract interfered with the interactions of the film components. Silva-Weiss et al. [24] also demonstrated that the *TS* of chitosan/corn starch films containing murta leaf extract decreased because of structural discontinuity and irregularity caused by the extract. Sago starch films containing betel leaves extract also showed similar results [25].

Regarding WVP, higher values were observed in ABS films containing CNE compared with that in films without CNE. These results are probably due to the weakened films by the incorporation of CNE, resulting in easy transfer of water molecules. Similarly, Assis et al. [18] demonstrated that the WVP of starch films was increased by the incorporation of β-carotene, which affected the diffusion of water vapor. Luchese et al. [26] also reported that the WVP of starch films containing blueberry pomace was increased because of decreased intermolecular interactions and increased mobility of the film components.

### 3.4. Color Measurement

With the addition of CNE to the ABS films, the *L* * value decreased whereas *a* *, *b* *, and Δ*E* increased (Table 2), indicating that the ABS film without CNE was highly transparent and colorless, whereas the films containing CNE had a slight red color. These results could be attributed to the natural pigment in CNE, which has a reddish color. In addition, with increasing amounts of CNE, a decrease in *L* * was observed, suggesting that the amount of CNE was associated with the lightness of the films. Cheng et al. [27] reported that films with low *L* * value could be prepared with the addition of phenolic compounds, and these films could protect packaged foods that are sensitive to light. Similarly, Talón et al. [28] reported that the incorporation of polyphenols from thyme extract into chitosan/starch films reduced the *L* * value of the films. Additionally, the opacity of ABS films containing CNE was higher than that of films without CNE. In general, the incorporation of active materials into the films increases the opacity of the films [18,23].

Similarly, Luchese et al. [26] demonstrated a decrease in lightness and an increase in the opacity of starch films containing blueberry pomace. Overall, the incorporation of CNE caused changes in the optical properties of the ABS films, especially the decrease in *L* * and increase in opacity. Therefore, the developed ABS films in this study are applicable to the packaging of fatty foods.

### 3.5. UV-Visible Light Transmittance

**Figure 2:**
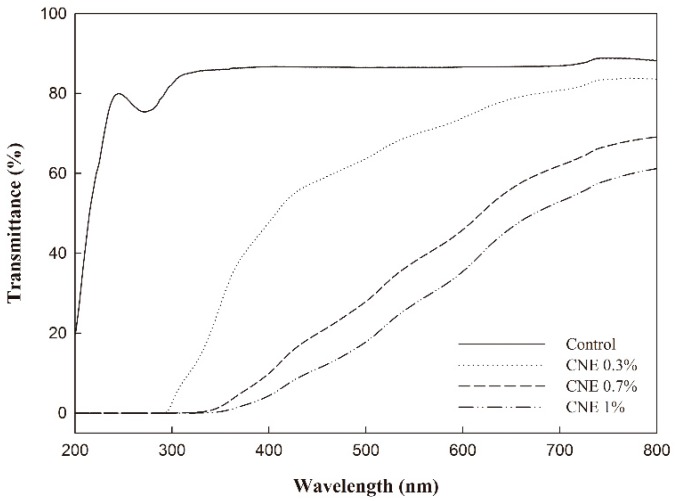
UV-visible light transmittance of adzuki bean starch films.

Figure 2 shows the UV-visible transmittance of the ABS films, where differences between the control (ABS film without CNE) and ABS films containing CNE were observed. In particular, the control film had high transmittance, whereas the ABS films containing CNE had relatively low transmittance (200 to 800 nm). Thus, the incorporation of CNE caused high absorption below 400 nm, corresponding to UV light, which could cause lipid oxidation in foods. In particular, the ABS films with 1% CNE prevented almost all UV transmission, indicating that the films could protect packaged foods against UV light [1]. Moreover, in the visible light range of 400 to 800 nm, the ABS films containing CNE had lower transmittance than that of the control film. Therefore, the ABS films containing CNE could be utilized as effective UV-shielding films. Similar results have been reported in starch films containing natural extracts [25,26].

### 3.6. TPC Analysis

**Figure 3:**
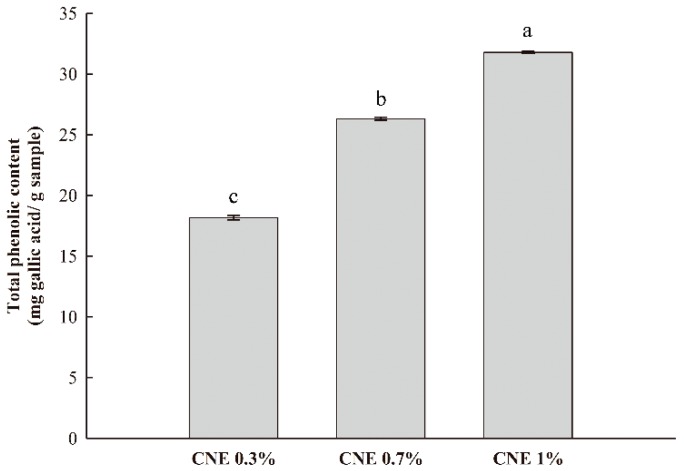
Total phenolic contents of adzuki bean starch films containing cocoa nibs extract.

The TPC of the ABS films containing CNE was determined (Figure 3). As expected, the TPC increased as the amount of CNE increased. In particular, the TPC of the ABS film with 1% CNE was 31.78 mg gallic acid/g film, which is relatively higher than previously reported values [29]. According to a report by Shojaee-Aliabadi et al. [30], the TPC of k-carrageenan films with 3% Satureja hortensis essential oil was 20.56 mg gallic acid/g film. In general, the TPC is related to antioxidant activity. Effective free radical scavenging capacity of natural extracts is mainly derived from phenolic constituents in their composition. Therefore, the antioxidant activity of the ABS films containing CNE is closely related with the TPC of the films.

### 3.7. Antioxidant Activity

As presented in Table 3, the ABS film without CNE did not exhibit antioxidant property, whereas the antioxidant property of the films containing CNE was proportional to the amount of added CNE. Similarly, Yang et al. [15] demonstrated that pure foxtail millet starch films had no antioxidant activity, but films with clove leaf oil showed increased ABTS and DPPH scavenging activities with increasing amounts of clove leaf oil. In this study, the ABS film containing 1% CNE showed the highest antioxidant activity, and the ABTS and DPPH scavenging activities were 100% and 94.9%, respectively. These results indicate that ABS films containing CNE have good antioxidant activity compared with that of antioxidant biodegradable films in other reports. Antioxidants can be released by molecular diffusion from the films to the packaged product. This phenomenon occurs within free volume of the polymer amorphous region. In addition, the stability of antioxidant activity of the films containing natural antioxidants is generally prolonged until 30 days. According to the report by Wu et al. [31], antioxidant capacity of the biodegradable films containing green tea extract showed stability during storage for 30 days. Therefore, ABS films containing CNE could prevent lipid oxidation of packaged foods and be applied as a potential active packaging material.

### 3.8. ATR-FTIR Analysis

**Figure 4:**
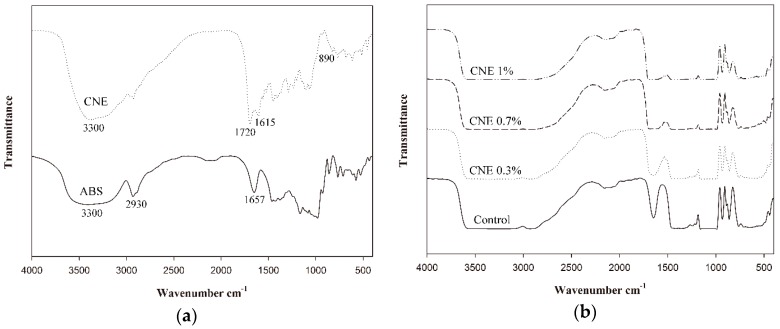
The attenuated total reflectance-Fourier transformation infrared (ATR-FTIR) spectra. (**a**) The components; (**b**) Adzuki bean starch films containing cocoa nibs extract.

The ATR-FTIR analysis was used to confirm the incorporation of CNE into the ABS films and examine the possible interactions between CNE and ABS in the films. The ATR-FTIR spectra of ABS, CNE, ABS films, and ABS films containing CNE are presented in Figure 4. The spectrum of ABS shows typical bands at 3300 cm^−1^ (OH stretching), 2930 cm^−1^ (CH stretching), and 1657 cm^−1^ (CO stretching) [6,7,23,26]. In contrast, CNE exhibited a peak at 1615 cm^−1^ (aromatic ring stretching), which was attributed to the phenolic constituents present in CNE. This peak was also observed in the spectrum of blueberry powder containing large amounts of polyphenols [26]. Additionally, at around 1720 cm^−1^, another peak appeared in the CNE spectrum, which was associated with the presence of C=O groups [26]. Meanwhile, the spectra of the ABS films were similar because of their similar compositions. As the amount of CNE increased, the absorbance at 3300 cm^−1^ (OH stretching) was shifted to 3150 cm^−1^, suggesting changes in hydrogen bonds and intermolecular distance in the composite films [6]. In addition, in the spectra of ABS films containing CNE, slight modifications were observed in the range of 1500 to 1700 cm^−1^, representing interactions between the starch chains and CNE [6,23]. Furthermore, the peak related to the CH_3_ rocking bands of ABS films was shifted from 862 cm^−1^ to higher wavenumbers with increasing CNE content, indicating the presence of chemical interactions between the compounds of CNE and ABS [6]. According to a report by Bof et al. [23], plant extracts containing polyphenolic compounds in the composition had a corresponding peak at 890 cm^−1^. Because CNE also showed a peak at 890 cm^−1^, this peak of the ABS films containing CNE became stronger with increasing CNE content and caused a shift of the band from 862 cm^−1^. In contrast, the absorbance at 1155 cm^−1^, which was assigned to C–O–C in glycosidic linkage, was similar regardless of the sample, suggesting that there was no change in the glycosidic chain length by the incorporation of CNE [7]. Overall, it should be noted that interactions occurred between ABS and CNE in the ABS films containing CNE, and these interactions altered the ATR-FTIR spectra.

### 3.9. DSC

**Figure 5:**
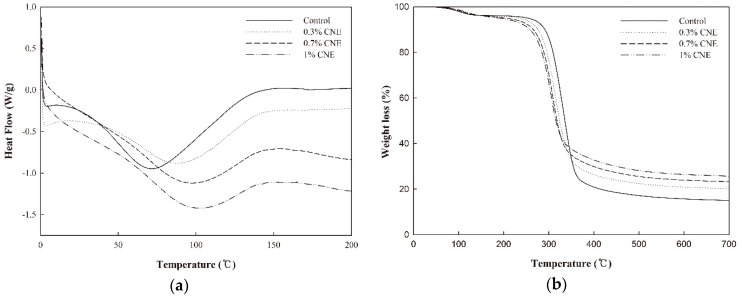
Thermal properties of adzuki bean starch films containing cocoa nibs extract. (**a**) Differential scanning calorimetry (DSC); (**b**) Thermogravimetric analysis (TGA).

DSC was carried out to analyze the thermal properties of the ABS films (Figure 5a, Table 4). Overall, the DSC results of the ABS films were similar to those reported in other studies [22,26]. In particular, the ABS films showed a broad endothermic peak (30 to 140 °C) regardless of the sample. The melting temperature (*T*_m_) of the ABS film (71.97 °C) was slightly lower than that of cassava starch films, which was 80 °C as reported by Jaramillo et al. [7]. Additionally, the *T*_m_ of the ABS films increased with increasing amounts of CNE, possibly because of the higher melting temperature of CNE than that of ABS. Similar results were reported for starch films with blueberry pomace [26] and thermoplastic starch films containing 3.76% thymol or 3.49% carvacrol [22]. In terms of enthalpy (Δ*H*), the ABS film without CNE had the highest value, suggesting that the addition of CNE decreased thermal energy to dissociate the films. These findings indicate that the thermal stability of the ABS films decreased with the addition of CNE because it increased polymer mobility, resulting in weakening of the film structure, as observed in the physical properties and FTIR of the films.

According to a report by Kuorwel et al. [22], the addition of active materials into the films could interfere with the association of polymer chains, causing a decrease in film crystallinity. Similarly, the Δ*H* of cassava starch films decreased because of changes in intramolecular interactions by the addition of polyphenol-rich compounds [7].

### 3.10. TGA

Figure 5b shows the thermal degradation of the ABS films assessed by TGA. In the present study, three typical weight loss stages were observed as reported in the literature [32]. The first stage (50 to 150 °C) was associated with water loss in the ABS films. The second stage was attributed to the decomposition of plasticizers added in the film-forming solution (150 to 220 °C). The third stage was detected in the range of 220 to 350 °C, corresponding to the degradation of ABS. In the third stage, a shift in degradation temperature was observed, suggesting that the ABS film without CNE was thermally stable compared with the films containing CNE. This decrease was reflected in the decrease in *TS* by the incorporation of CNE in the films, which was mainly due to the structural change in the film network. These findings are consistent with the DSC results, where the addition of CNE decreased the thermal stability of the films. Regarding this phenomenon, there have been many similar reports [33]. In addition, the final weights of ABS, ABS-0.3% CNE, ABS-0.7% CNE, and ABS-1% CNE films were 15.02%, 20.21%, 23.25%, and 25.71% of the original weight, respectively.

### 3.11. Biodegradability Test

To determine the biodegradability of the ABS films, the weight loss of the films buried in vegetable compost was measured for 28 days (Table 5). All films showed changes in weight after 4 days, indicating the beginning of degradation. With the incorporation of CNE, the weight loss of the ABS films increased, and all films were almost completely decomposed after 4 weeks. This phenomenon could be attributed to the moisture and enzymatic activity of microorganisms in the soil [33]. Similar to these results, Xiong et al. [34] demonstrated that the complete decomposition of starch films occurred after 100 days. Torres et al. [35] also reported that the weight loss of starch films buried in soil increased to 99.35% after one month of storage. It should be noted that the biodegradability of the ABS films was improved by the addition of CNE to the films. This improvement could be explained as CNE contained certain compounds with low molecular weight, which could degrade before ABS. Another explanation is that film biodegradability can be associated with the water solubility of the films. High water solubility leads to fast polymer disintegration during biodegradation [18]. In this study, the ABS films showed an average solubility of 32% and the solubility increased with the addition of CNE (Table 6), resulting in the high biodegradability of the ABS films containing CNE.

## 4. Conclusions

Biodegradable films based on starch extracted from adzuki bean were developed, and CNE was incorporated to confer antioxidant activity to the films in this study. The incorporation of CNE increased the UV-shielding effect of the films and resulted in good radical scavenging activity against ABTS and DPPH. Moreover, the ABS films containing CNE exhibited higher biodegradability than that of films without CNE. These results suggest that ABS films containing CNE can be applied as a new active packaging material in the food industry.

## Figures and Tables

**Table 1:** Physical properties of adzuki bean starch (ABS) films containing various amounts of cocoa nibs extract (CNE).

CNE (%)	Thickness (mm)	Tensile Strength (MPa)	Elongation at Break (%)	Water Vapor Permeability (10^−9^ g /m s Pa)
0	0.053 ± 0.002 ^d^	30.59 ± 2.21 ^a^	10.11 ± 2.30 ^d^	2.11 ± 0.12 ^b^
0.3	0.058 ± 0.001 ^c^	27.74 ± 0.98 ^b^	13.81 ± 0.90 ^c^	2.42 ± 0.27 ^ab^
0.7	0.062 ± 0.001 ^b^	25.50 ± 1.12 ^c^	24.51 ± 2.51 ^b^	2.55 ± 0.30 ^ab^
1	0.066 ± 0.003 ^a^	18.58 ± 1.60 ^d^	27.80 ± 2.46 ^a^	2.81 ± 0.49 ^a^

Mean ± SD. ^a–d^ Any means in the same column followed by different letters are significantly (*p* < 0.05) different by Duncan’s multiple range test.

**Table 2:** Optical properties of ABS films containing various amounts of cocoa nibs extract.

CNE (%)	*L* *	*a* *	*b* *	∆*E*	Opacity (A/mm)
0	95.93 ± 0.50 ^a^	−0.22 ± 0.03 ^d^	2.31 ± 0.26 ^d^	-	1.34 ± 0.10 ^d^
0.3	88.69 ± 0.13 ^b^	1.94 ± 0.07 ^c^	14.48 ± 0.12 ^c^	14.32 ± 0.06 ^c^	2.02 ± 0.11 ^c^
0.7	75.99 ± 0.18 ^c^	6.94 ± 0.08 ^b^	30.92 ± 0.26 ^b^	35.66 ± 0.28 ^b^	4.84 ± 0.03 ^b^
1	69.49 ± 0.86 ^d^	10.97 ± 0.50 ^a^	36.62 ± 0.45 ^a^	44.91 ± 0.87 ^a^	5.63 ± 0.08 ^a^

Mean ± SD. ^a–d^ Any means in the same column followed by different letters are significantly (*p* < 0.05) different by Duncan’s multiple range test.

**Table 3:** Antioxidant activities of ABS films containing various amounts of cocoa nibs extract.

CNE (%)	ABTS Radical Scavenging (%)	DPPH Radical Scavenging (%)
0	-	-
0.3	97.70 ± 0.86 ^b^	66.13 ± 0.21 ^c^
0.7	99.72 ± 0.24 ^a^	94.22 ± 0.40 ^b^
1	100.00 ± 0.00 ^a^	94.88 ± 0.05 ^a^

Means ± SD. ^a–c^ Any means in the same column followed by different letters are significantly (*p* < 0.05) different by Duncan’s multiple range test.

**Table 4:** Thermal properties of ABS films containing various amounts of cocoa nibs extract.

CNE (%)	*T*_m_ (°C)	Δ*H* (J/g)
0	71.97	199.51
0.3	87.69	129.52
0.7	96.81	146.50
1	102.29	110.48

**Table 5:** Biodegradability of ABS films containing various amounts of cocoa nibs extract.

CNE (%)	Weight Loss (%)				
	4 d	7 d	14 d	21 d	28 d
0	22.58 ± 0.23 ^Ec^	42.27 ± 1.93 ^Dc^	58.12 ± 1.56 ^Cc^	75.52 ± 2.05 ^Bc^	98.28 ± 0.52 ^Ab^
0.3	26.75 ± 1.09 ^Eb^	51.32 ± 2.11 ^Db^	67.84 ± 2.50 ^Cb^	93.38 ± 0.66 ^Bb^	99.26 ± 0.35 ^Aa^
0.7	28.41 ± 1.67 ^Eb^	52.14 ± 2.50 ^Dab^	74.25 ± 2.66 ^Ca^	96.07 ± 1.09 ^Ba^	99.57 ± 0.24 ^Aa^
1	34.41 ± 0.59 ^Ea^	56.05 ± 1.98 ^Da^	75.89 ± 1.28 ^Ca^	97.45 ± 0.63 ^Ba^	99.76 ± 0.24 ^Aa^

Means ± SD. ^a–c^ Any means in the same column followed by different letters are significantly (*p* < 0.05) different by Duncan’s multiple range test. ^A–E^ Any means in the same row followed by different letters are significantly (*p* < 0.05) different by Duncan’s multiple range test.

**Table 6:** Water solubility and moisture content of ABS films containing various amounts of cocoa nibs extract.

CNE (%)	Water Solubility (%)	Moisture Content (%)
0	32.38 ± 1.09 ^d^	7.25 ± 0.33 ^b^
0.3	35.25 ± 0.41 ^c^	7.90 ± 0.80 ^ab^
0.7	39.77 ± 0.52 ^b^	8.37 ± 0.31 ^a^
1	44.30 ± 0.54 ^a^	8.43 ± 0.34 ^a^

Means ± SD. ^a–d^ Any means in the same column followed by different letters are significantly. (*p* < 0.05) different by Duncan’s multiple range test.
